# *PolSpec* : Polarisation‐Based Detection for Versatile, Cost‐Effective Rapid Hyperspectral Imaging

**DOI:** 10.1002/jbio.70012

**Published:** 2025-03-24

**Authors:** Huihui Liu, Sunil Kumar, Edwin Garcia, Darren Ennis, Iain A. McNeish, Paul M. W. French

**Affiliations:** ^1^ Physics Department Imperial College London London UK; ^2^ Francis Crick Institute London UK; ^3^ Department of Surgery & Cancer Hammersmith Hospital, Imperial College London London UK

**Keywords:** hyperspectral imaging, polarisation, spectral classification, spectral unmixing

## Abstract

“*PolSpec*” is a flexible, cost‐effective approach for rapid (including single‐shot) spectrally resolved imaging. While established approaches, e.g., using cascades of dichroic beamsplitters, diffractive image splitters, or mosaic filters, typically have pre‐determined spectral detection bands with cost and experimental complexity scaling with the number of spectral channels, *PolSpec* uses polarisation optics to provide continuously varying transmission across a configurable spectral range to generate “spectral modulation vectors” that can represent specific spectral signatures with lower data volumes than full spectral profiles. It can be implemented with almost any detector. Here we demonstrate low‐cost single‐shot widefield *PolSpec*‐based hyperspectral imaging using a polarisation‐resolving camera.

## Graphical abstract

“*PolSpec*” is a fast, flexible, data efficient and cost‐effective technique for hyperspectral imaging utilising polarisation‐based spectral modulation. Using data‐efficient representation of spectral information on vector plots, it facilitates rapid visualisation and segmentation of spectral contrast to classify sample features. As a proof of concept, a single‐shot widefield *PolSpec* module was implemented using a polarisation‐resolving camera and applied to imaging H&E‐stained histological sections and fluorescent pollen grain samples.

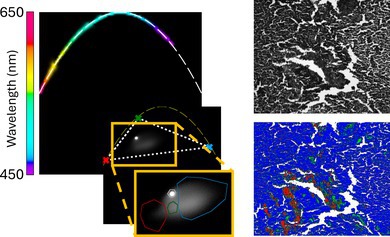

**Abbreviations**
AQWP: achromatic quarter wave plate
FOV: field of view
IRF: instrument response function
LCR: liquid crystal variable retarder
*PolSpec*
: polarisation‐resolved spectral modulation
SMV: spectral modulation vector
SNR: signal‐to‐noise ratio

## 1 Introduction

Spectrally resolved imaging has been implemented with almost every optical imaging modality, from endoscopy to microscopy and tomography, where spectroscopic information can provide molecular contrast, which is often combined with spatial information in images to identify/classify different states of a sample. It has a wide range of applications, including cell and tissue pathology for clinical diagnosis, biochemical assays, spatial proteomics; brightfield and fluorescence imaging of plants, e.g., to assess stress, crop ripeness, or the impact of chemical agents (agrochemicals, pollutants), environmental monitoring and quality assurance of optoelectronic devices such as solar cells or displays, and other diverse research applications. Multispectral and hyperspectral imaging techniques are also widely used to distinguish and/or unmix light from different chromophores with overlapping spatial distributions in a range of applications. Increasingly, spectrally resolved imaging is used with machine learning to, e.g., increase performance or automate identification/classification processes, e.g., [1, 2, 3].

Multispectral imaging usually refers to approaches where a relatively small number of discrete spectral channels are resolved and is commonly implemented using a mosaic spectral mask (e.g., RGB camera) or by sequential image acquisitions through either a set of spectral filters or a single electronically tuneable bandpass spectral filter, such as a liquid crystal tuneable filter (LCTF) or an acousto‐optic tuneable filter (AOTF). The use of spectral filters is inherently lossy when out‐of‐band light is rejected. More efficient—and “single‐shot”—multispectral imaging can be implemented for a (relatively small) number of spectral channels using (cascaded) dichroic beamsplitters with multiple detectors—or using a spectrally resolving image splitter with a single camera to simultaneously acquire image data in different spectral passbands.

Hyperspectral imaging refers to techniques that resolve signals with respect to a continuous set of adjacent (typically ≫ 10) spectral channels (bins) so that a “full” spectral profile is acquired for each image pixel. Hyperspectral datasets can also be acquired with sequential image acquisitions via a scanned narrow band filter (typically a LCTF or AOTF), although this is photon inefficient, with the loss increasing with the spectral resolution. For more photon efficient hyperspectral imaging, point scanning (“whisk”) or line‐scanning (“push‐broom”) instruments can direct signal light to a spectrometer or spectrograph to capture full spectra for the pixels interrogated, in principle, with no out‐of‐band loss. However, the need to scan along at least one spatial dimension slows down the hyperspectral image data acquisition.

For many implementations, hyperspectral imaging acquires more information than is needed for applications such as spectral classification and/or unmixing. Large hyperspectral datasets are often compressed for these purposes using techniques like principal component analysis (PCA) [4], tensor decomposition [5], or spectral phasor analysis [6, 7, 8] which utilises the Fourier transform of the spectral data. Spectral phasor analysis typically entails multiplying the acquired hyperspectral image data with sine and cosine functions and integrating them along the spectral dimension to generate vectors on a polar plot (“phasor plot”) that are often referred to as “spectral phasors.” These vectors represent specific spectral characteristics (e.g., the mean wavelength and the width of spectra) at each image pixel. Where multiple spectral components are present, the spectral phasor is the linear sum of the component phasors weighted by their respective proportions. If the component (“reference”) spectra are known a priori, a spectral phasor can be “unmixed” to give the proportions of each spectral phasor component. In principle, spectral phasors calculated by sine and cosine modulation functions at a single frequency can support linear unmixing of up to three spectral components; unmixing more spectral components requires calculation with higher harmonics of the modulation frequency used. Spectral phasor plots can provide convenient visualisations of hyperspectral image data—often enabling different spectral components distributed across a field of view (FOV) to be discerned without further calculations—and this compact (reduced) form of hyperspectral data is amenable to diverse computational and graphical analysis approaches.

Historically, spectral phasor analysis has primarily been applied computationally to “full” (x–y–λ) hyperspectral datasets, which suggests that more information may have been acquired (and more photons detected) than necessary for the intended application. Recently, however, the direct acquisition of spectral phasor data, e.g., [9, 10], by acquiring widefield images transmitted/reflected through filters/dichroics with sinusoidally varying spectral transmission functions is attracting interest: this approach produces much smaller data footprints compared to conventional hyperspectral imaging, provides faster acquisition speeds and/or higher signal‐to‐noise ratios (SNR) compared to approaches that spread incident photons over many spectral channels, and is compatible with diffuse optical signals. The implementation reported by Dvornikov and Gratton [9] sequentially acquired three transmitted wide‐field images: two using sinusoidal and cosinusoidal modulating spectral filters and a third image acquired with no spectral filters, to calculate the spectral phasors across the image. This implementation loses the photons that are not transmitted through the filters, and the sequential acquisitions required mechanical filter changes, limiting the acquisition speed. Subsequently, Wang et al. [10] demonstrated a more efficient single‐shot implementation, “SHy‐Cam” using two customised dichroics with sinusoidal and cosinusoidal spectral transmission functions, respectively. A four‐channel image‐splitter was used to simultaneously capture four widefield images transmitted and reflected from these two special dichroics. This implementation for direct acquisition of spectral phasor data is nominally lossless with respect to the spectral filtering, but the set‐up is relatively cumbersome and expensive.

In the above demonstrations of direct phasor generation, Dvornikov and Gratton identified off‐the‐shelf filters for their implementation, while the dichroic beamsplitters used in the SHy‐Cam were custom‐made. Adjusting the operational parameters of these direct spectral phasor imaging implementations, such as the spectral range, resolution and higher harmonics of sinusoidal modulation functions, requires changing filters and/or dichroic beamsplitters, which may require new dielectric coatings to be designed and fabricated, incurring significant costs. To address the issue of flexibility and to efficiently acquire the information needed for spectral classification and unmixing with reduced costs and minimal experimental complexity, here we propose “*PolSpec*” where we utilise polarisation optics to implement hyperspectral imaging using “spectral modulation vectors” (SMVs), a generalised approach that includes but is not limited to spectral phasor imaging (where sine/cosine modulation functions are used and discussed in terms of Fourier transforms). SMV analysis can be used with any convenient sets of continuous orthogonal modulation functions, offering opportunities to reduce experimental complexity.

*PolSpec* utilises common polarisation optics, i.e., linear polarisers, polarising beamsplitters, birefringent optical retarders, etc., instead of (bespoke) spectral filters/beamsplitters, to implement spectral modulation functions using adaptations of the Lyot filter [11] and thus realise the direct acquisition of SMVs. A Lyot filter provides spectrally varying transmission for light that is passed through an appropriately orientated optical retarder placed between linear polarisers. This forms an interferometer such that when the axes of the linear polarisers are aligned, light at wavelengths experiencing a phase delay of integer multiples of 2*π* between the ordinary and extraordinary axes of the retarder will constructively interfere. Light at other wavelengths will be attenuated according to the law of Malus with a cosine dependence on frequency, as discussed below.

This approach to generating spectral modulation functions can widen access to hyperspectral imaging through the wide availability of low‐cost polarisation optics, including polymer linear polarising and retarder (quarter and half wave) sheets that can be cut to fabricate components as desired. Alternatively, electronically configurable liquid crystal variable retarders (LCRs) may be used for fast tuning of operational parameters. Here we present a low‐cost single‐shot implementation of PolSpec that utilises a polarisation‐resolving (Polarsens) CMOS camera to realise a compact hyperspectral imaging module, which we have implemented on a widefield epifluorescence microscope. As a proof of concept, the instrument response function (IRF) was measured using a tuneable supercontinuum laser source, and the hyperspectral imaging functionality was applied to brightfield imaging of histological sections and fluorescence from pollen grain samples.

We note that combinations of polarising optical components and retarders have previously been configured to provide photon‐efficient single‐shot multispectral imaging instruments (i.e., dividing incident photons between discrete spectral bins), e.g., [12], but this approach entails increasing complexity as the number of spectral bins is increased, requires spatially separate detection sensor regions of the camera(s) for each spectral channel, and does not directly generate the signals required to calculate the SMVs. We further note that computational optimisation has been used to design specific combinations of polarising optical components and retarders with a polarisation‐resolving camera to provide photon‐efficient spectral filters for detection of specific spectral features, e.g., gas absorption lines [13]. In contrast to [12, 13] where the optical configurations are optimised for predetermined spectral properties, our SMV approach is designed to be spectrally agnostic, able to capture hyperspectral image information across the spectral range supported by the camera.

## 2 Methods

### 2.1 Generalised Spectral Modulation Vector (SMV)

For an incident spectrum $I_{i}\left(\nu \right)$, a N‐dimensional (normalised) SMV, $\vec{V}=\left(V_{1},V_{2},\ldots ,V_{N}\right)$, can be calculated by using a set of N different spectral modulation functions, $\left\{P_{k}\left(\nu \right)\right\}$ where k=1,2,…N, and following

(1) $$V_{k}=\frac{\int I_{i}\left(\nu \right)\cdot P_{k}\left(\nu \right)\cdot \mathrm{d\nu}}{\int I_{i}\left(\nu \right)\cdot \mathrm{d\nu}},\;k=1,2,\ldots N$$

For the convenience of linear analysis, any two different spectral modulation functions used should be orthogonal, i.e.,

(2) $$\int P_{l}\left(\nu \right)\cdot P_{m}\left(\nu \right)\cdot \mathrm{d\nu}\overset{l\neq m}{=}0,\;l,m\in \left[1,2,\ldots ,N\right]$$

Note that the frequency ν in Equations (1) and (2) can also be substituted by the wavelength λ, if preferred in practical implementations.

Spectral phasors are specific instances of SMVs generated by $\left\{\cos\left(\mathrm{nf\lambda}\right),\sin\left(\mathrm{nf\lambda}\right)\right\}$ spectral modulation functions, where f is the base frequency and n is the harmonic number, which is selected as needed in practise. The concept of SMVs extends the choice of spectral modulation functions generally to sets of continuous orthogonal functions, retaining the advantages of effective data reduction, e.g., for spectral classification or unmixing applications, while broadening the range of convenient/practical implementations for direct acquisition of hyperspectral SMV image data.

### 2.2 Polarisation‐Based Spectral Modulation ( *PolSpec* )

**FIGURE 1:**
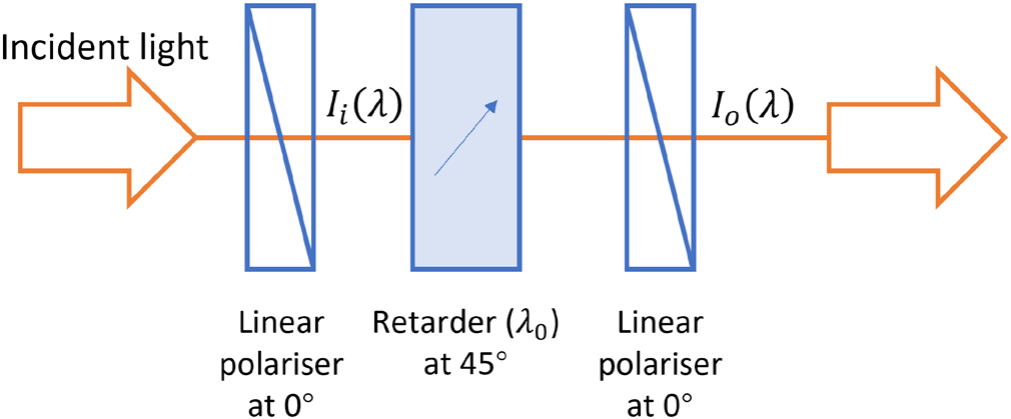
Schematic of a Lyot filter.

For direct acquisition of hyperspectral SMV image data, the required orthogonal spectral modulation functions can be conveniently realised using polarisation optics: *PolSpec* is based on the well‐known concept of Lyot filters [11], in which a retarder is usually placed between two parallel (or orthogonal) linear polarisers with its ordinary/extraordinary axes orientated at 45° to the fast/slow axes of the linear polarisers. Figure 1 depicts a configuration for a Lyot filter, whose spectral transmission function $T\left(\nu \right)$ or $T\left(\lambda \right)$ for normally incident light is given by the ratio of the output intensity $I_{o}$ to the input intensity $I_{i}$ as a function of wavelength λ (or frequency ν):

(3) $$T\left(\lambda \right)=\frac{I_{o}\left(\lambda \right)}{I_{i}\left(\lambda \right)}=\cos^{2}\left(\frac{\pi \lambda_{0}}{\lambda }\right)=\frac{1}{2}\left[1+\cos\left(\frac{2\pi \lambda_{0}}{\lambda }\right)\right],\;\lambda_{0}=\mathrm{\delta n}\cdot d$$

(4) $$\Rightarrow T\left(\nu \right)\propto 1+\cos\left(\Lambda \nu \right),\;\Lambda =\frac{2\pi \lambda_{0}}{c},\;\lambda \nu =c$$

where ∝ represents proportionality and c denotes the speed of light in free space. $\lambda_{0}$ denotes the retardance, i.e., optical path difference between the extraordinary and ordinary axes, of the retarder. δn denotes the refractive index difference between the extraordinary and ordinary axes of the retarder, and d denotes the thickness of the retarder.

From Equation (4), it is straightforward to configure retarder(s) and linear polarisers to achieve a cosine transmission function. Varying the retardance $\lambda_{0}$ of the retarder(s) results in a change of frequency Λ of the cosine function, which consequently changes the spectral modulation function. If conventional spectral phasor analysis is desired, the required sine modulation function requires a frequency‐independent quarter wave (*π*/2) phase shift of the transmission function, which cannot be realised by simply adjusting the retardance. This can be achieved by inserting an achromatic quarter wave plate (AQWP) before or after the retarder. However, AQWPs are expensive and may not be available for the desired operating spectral range. Therefore, instead of aiming to generate cosine/sine spectral modulation functions for phasor analysis, we instead generate SMVs using a different set of orthogonal spectral modulation functions, $\left\{\cos\left(\mathrm{k\Lambda \nu}\right)\right\}$ where k=1,2,…, which are much easier to implement using low‐cost polarisation optics.

### 2.3 Single‐Shot *PolSpec* Configurations

**FIGURE 2:**
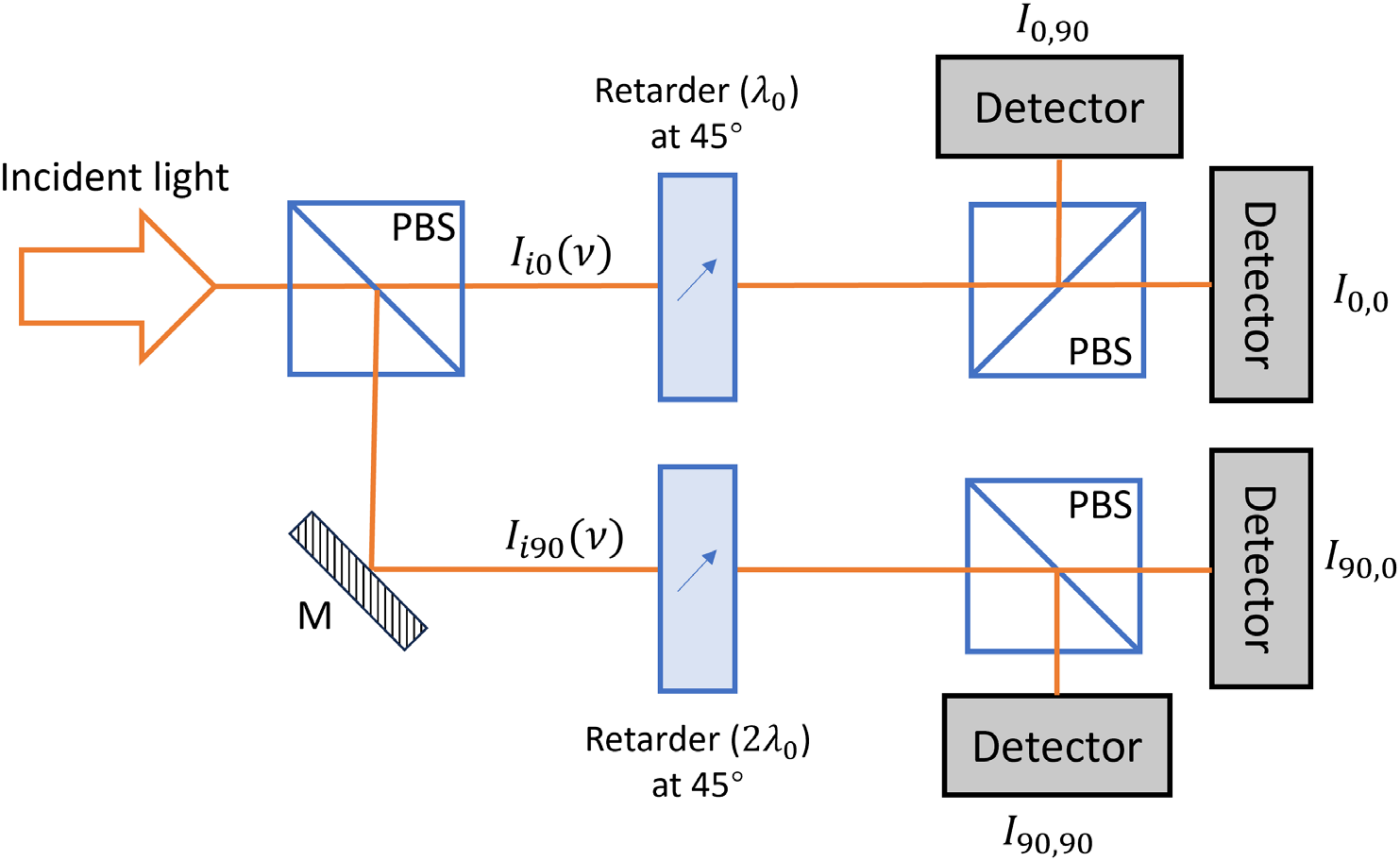
Single‐shot *PolSpec* with $\left\{\cos\left(\Lambda \nu \right),\cos\left(2\Lambda \nu \right)\right\}$ spectral modulation functions.

A single‐shot *PolSpec* configuration analogous to the SHy‐Cam [10] is shown in Figure 2. The incident light is first divided into two orthogonally linearly polarised beams, $I_{i0}$ and $I_{i90}$. Each beam then transmits through a retarder with a retardance of $\lambda_{0}$ or $2\lambda_{0}$, respectively, with the extraordinary/ordinary axes oriented at 45° to the respective beam polarisation. After that, each beam is further split into another two orthogonally linearly polarised beams, both of which are then detected as the complement of each other. Effectively the input light is passed through two Lyot filters in parallel, where both the transmitted and reflected light are detected at the output polarisers of these birefringence‐based interferometers.

The readouts from four detectors in Figure 2 are respectively:

(5) $$I_{0,0}=\int \frac{I_{i0}\left(\nu \right)}{2}\left[1+\cos\left(\Lambda \nu \right)\right]\cdot \mathrm{d\nu},\;I_{0,90}=\int \frac{I_{i0}\left(\nu \right)}{2}\left[1-\cos\left(\Lambda \nu \right)\right]\cdot \mathrm{d\nu}$$

(6) $$I_{90,0}=\int \frac{I_{i90}\left(\nu \right)}{2}\left[1-\cos\left(2\Lambda \nu \right)\right]\cdot \mathrm{d\nu},\;I_{90,90}=\int \frac{I_{i90}\left(\nu \right)}{2}\left[1+\cos\left(2\Lambda \nu \right)\right]\cdot \mathrm{d\nu}$$

Therefore, 2D SMVs corresponding to the spectral modulation functions $\left\{\cos\left(\Lambda \nu \right),\cos\left(2\Lambda \nu \right)\right\}$ can be generated by

(7) $$\begin{array}{c} \vec{V}=\left(\frac{I_{0,0}-I_{0,90}}{I_{0,0}+I_{0,90}},\frac{I_{90,90}-I_{90,0}}{I_{90,90}+I_{90,0}}\right)\\ \;=\left(\frac{\int I_{i0}\left(\nu \right)\cdot \cos\left(\Lambda \nu \right)\cdot \mathrm{d\nu}}{\int I_{i0}\left(\nu \right)\cdot \mathrm{d\nu}},\frac{\int I_{i90}\left(\nu \right)\cdot \cos\left(2\Lambda \nu \right)\cdot \mathrm{d\nu}}{\int I_{i90}\left(\nu \right)\cdot \mathrm{d\nu}}\right) \end{array}$$

This configuration is single‐shot, in principle lossless, and can also provide information about polarisation anisotropy of the incident light. However, it divides the incident beam into four paths in a similar manner to the SHy‐Cam [10], increasing the optomechanical complexity, and requiring four detectors.

**FIGURE 3:**
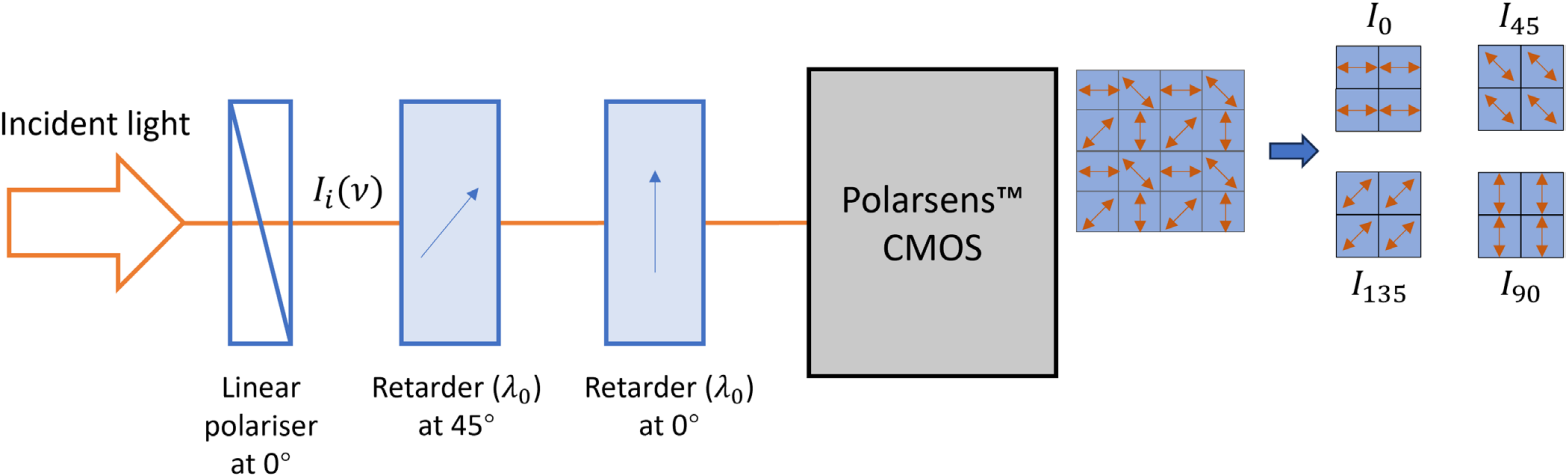
Polarsens‐based single‐shot *PolSpec* with $\left\{\cos\left(\Lambda \nu \right),\sin^{2}\left(\Lambda \nu \right)\right\}$ spectral modulation functions.

A more compact and low‐cost configuration of single‐shot *PolSpec* can be realised using a polarisation‐resolving camera sensor (“Polarsens” [14]), as shown in Figure 3. A Polarsens sensor incorporates a pixel‐wise linear polariser mask with fast axes in groups of 2×2 pixels orientated at 0°, 45°, 135° and 90°, such that a single shot effectively outputs four polarisation‐resolved sub‐images, $I_{0}$, $I_{45}$, $I_{135}$ and $I_{90}$, as if they had been imaged through linear polarisers with fast axes at these orientations.

The sub‐images from the Polarsens camera in the set‐up depicted in Figure 3 can be respectively written as:

(8) $$I_{0}=\int \frac{I_{i}\left(\nu \right)}{2}\left[1+\cos\left(\Lambda \nu \right)\right]\cdot \mathrm{d\nu},\;I_{90}=\int \frac{I_{i}\left(\nu \right)}{2}\left[1-\cos\left(\Lambda \nu \right)\right]\cdot \mathrm{d\nu}$$

(9) $$I_{45}=\int \frac{I_{i}\left(\nu \right)}{4}\left[3-\cos\left(2\Lambda \nu \right)\right]\cdot \mathrm{d\nu},\;I_{135}=\int \frac{I_{i}\left(\nu \right)}{4}\left[1+\cos\left(2\Lambda \nu \right)\right]\cdot \mathrm{d\nu}$$

Therefore, 2D SMVs corresponding to the spectral modulation functions $\left\{\cos\left(\Lambda \nu \right),\sin^{2}\left(\Lambda \nu \right)\right\}$ can be generated by:

(10) $$\begin{array}{c} \vec{V}=\left(\frac{I_{0}-I_{90}}{I_{0}+I_{90}},\frac{I_{45}-I_{135}}{I_{45}+I_{135}}\right)\\ \;=\left(\frac{\int I_{i}\left(\nu \right)\cdot \cos\left(\Lambda \nu \right)\cdot \mathrm{d\nu}}{\int I_{i}\left(\nu \right)\cdot \mathrm{d\nu}},\frac{\int I_{i}\left(\nu \right)\cdot \sin^{2}\left(\Lambda \nu \right)\cdot \mathrm{d\nu}}{\int I_{i}\left(\nu \right)\cdot \mathrm{d\nu}}\right) \end{array}$$

Note that $\sin^{2}\left(\Lambda \nu \right)=\frac{1-\cos\left(2\Lambda \nu \right)}{2}$ is essentially still a cosine function.

This compact configuration only requires one linear polariser and two retarders, which can be cut from low‐cost polymer sheets, and the Polarsens camera itself is approximately a cube of side 3 cm weighing 36 g [14]. Such a compact design is suitable for applications with space and/or weight constraints, e.g., on a drone for aerial surveillance or in a handheld device for point‐of‐care diagnostics. In principle, it may be possible to integrate thin film polarisers and retarders directly in front of the Polarsens sensor chip. SI Figure S1 presents an example of brightfield imaging of a colour test chart using the apparatus depicted in Figure 3.

For specific applications, the compact size and low cost of this configuration should be considered in the context of the imaging performance of the Polarsens camera, which is an uncooled CMOS camera designed for machine vision applications that may present too much thermal noise for some scientific applications. Furthermore, the pixel‐wise polariser mask in front of the Polarsens sensor blocks half the light incident, reducing the detection photon efficiency by 50%. This camera exhibits high sensitivity across the visible spectral range, but its polarisation extinction ratio decreases significantly with increasing wavelength from 450 nm, dropping to 100:1 at ~700 nm [14].

### 2.4 Experimental Polarsens‐Based Single‐Shot *PolSpec* Implementation

**FIGURE 4:**
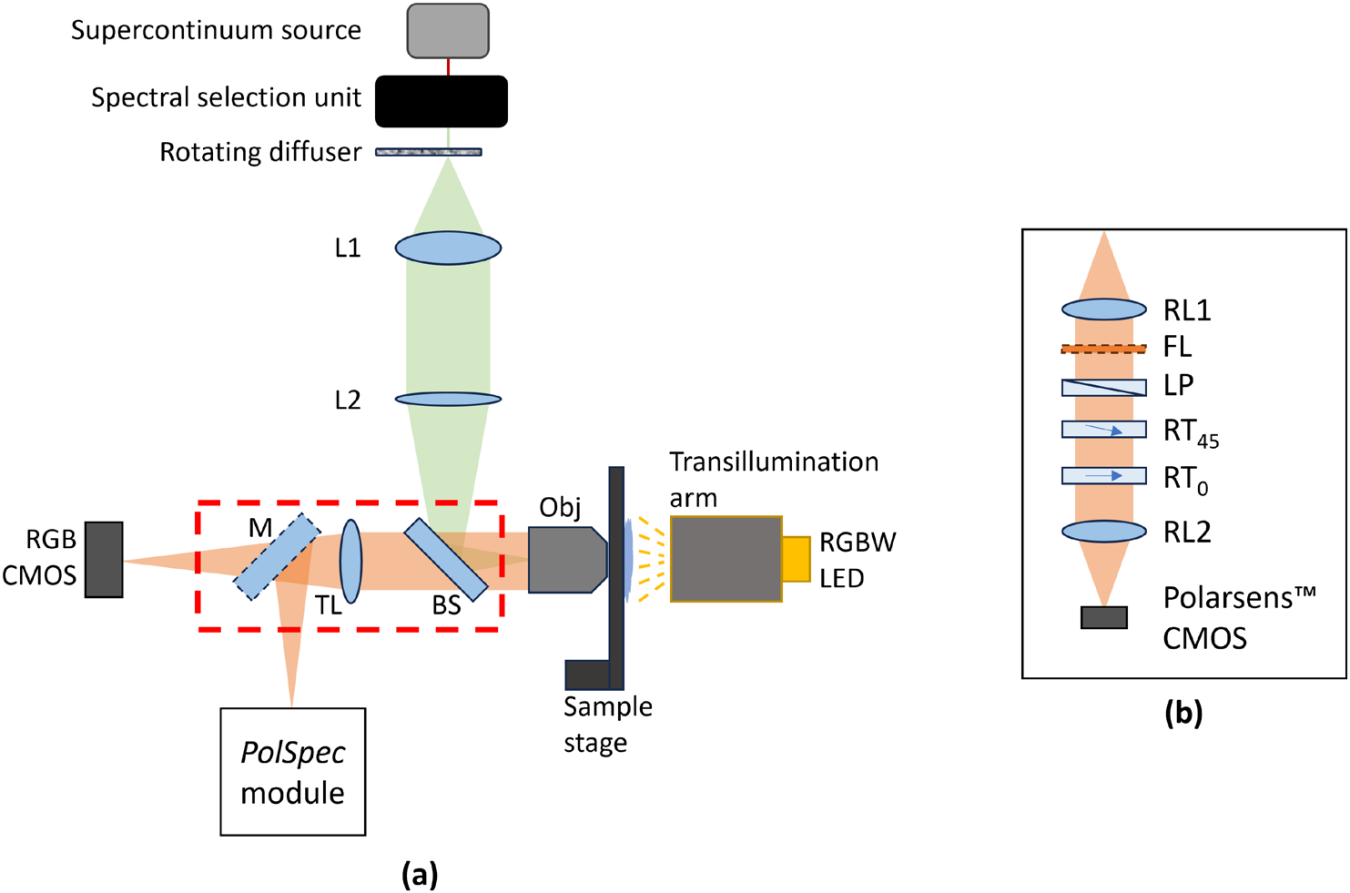
System diagrams of (a) an Olympus IX71 microscope frame with (b) a Polarsens‐based single‐shot *PolSpec* module implemented. (L1 and L2: Lenses of 50 and 300 mm focal lengths respectively; BS: 50:50 beamsplitter; Obj.: Objective lens; TL: Tube lens; M: Steering mirror; RL1 and RL2: Relay lenses of 100 mm focal lengths; FL: Emission filter for fluorescence imaging; LP: Linear polariser; RT_45_ and RT_0_: Retarders with extraordinary axes oriented at 45° and 0°, respectively).

The *PolSpec* configuration shown in Figure 3 was implemented as an add‐on module to an existing microscope frame (Olympus IX71) previously configured for widefield epifluorescence and brightfield imaging. The system diagrams of the microscope with the implemented *PolSpec* module are presented in Figure 4.

For widefield epifluorescence, the microscope utilised a tuneable excitation source [15] based on an optical fibre laser‐based supercontinuum (Fianium, WhiteLase SC400‐4). The broadband output from the supercontinuum source was bandpass filtered by a prism‐based spectral selection unit [15], which provides computer control of the centre wavelength and bandwidth of the excitation light. This excitation beam was focused onto a rotating diffuser that was imaged to the objective pupil plane by two lenses, L1 (Thorlabs, AC254‐050‐A) and L2 (Thorlabs, AC254‐300‐A), to realise Köhler illumination for epifluorescence microscopy. For brightfield imaging, the original light source, a halogen lamp, in the transillumination arm of the microscope frame was replaced by an RGBW LED module (iPixel LED Light Co. Ltd., P032001MA4SD6) to provide Köhler transillumination with computer‐controlled colour and brightness.

Florescence emission or transilluminated light from the sample was collected by a 10× objective lens, Obj. (Olympus, UPlanFLN 10× 0.3NA), then transmitted through a 50:50 beam splitter, BS, which served as a “broadband dichroic” beamsplitter and a tube lens embedded inside the microscope frame (red dashed bounded box in Figure 4a), and eventually directed by a steering mirror, M, to either a conventional RGB CMOS camera (Cairn Research Ltd., CellCam Centro 200MR) for brightfield imaging or the *PolSpec* module (black solid bounded boxes in Figure 4) for brightfield or epifluorescence (hyperspectral) imaging.

The *PolSpec* module was mounted at a camera port of the microscope frame, with two identical lenses, RL1 and RL2 (Thorlabs, AC254‐100‐A), relaying the image onto a Polarsens camera (Teledyne FLIR, Blackfly BFS‐U3‐51S5P‐C). All polarisation optics were placed in the infinity space of this image relay: the linear polariser, LP, was cut from a low‐cost polymer polarising film (Edmund Optics, XP42‐40) and the retarders, RT_45_ and RT_0_, were standard half wave plates designed for 670 nm (Thorlabs, WPH10E‐670). For epifluorescence, a long pass filter, FL, cutting off at a wavelength longer than the excitation band, was selected as appropriate and inserted between RL1 and LP as the emission filter.

## 3 Results

### 3.1 Mapping the SMV Instrument Response Function (IRF)

**FIGURE 5:**
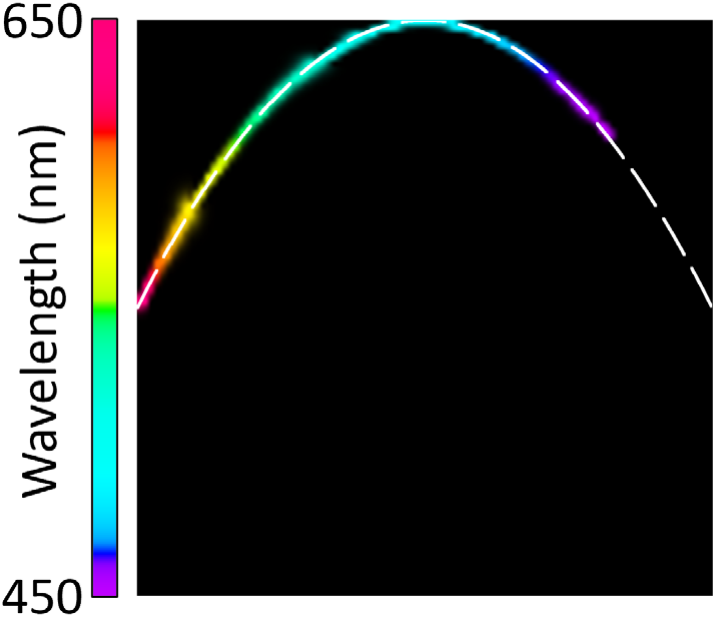
“Rainbow response curve” mapping the hyperspectral IRF of the Polarsens‐based single‐shot *PolSpec* module between 450 nm and 650 nm. The white dashed line indicates the respective theoretical IRF curve for the *PolSpec* configuration with $\left\{\cos\left(\Lambda \nu \right),\sin^{2}\left(\Lambda \nu \right)\right\}$ spectral modulation functions.

The spectral selection unit after the supercontinuum source was set to provide a collimated beam with a spectral bandwidth of less than 3 nm and a centre wavelength tuneable between 450 and 650 nm. This beam was then directed via a mirror in the sample plane of the microscope to the entrance of the *PolSpec* module. By tuning the centre wavelength of the incident beam, which approximates a spectral delta function, the SMVs obtained can be mapped out as a function of centre wavelength to provide the IRF of this *PolSpec* module. This is illustrated in Figure 5 where SMVs generated are coloured with their respective wavelengths to produce a “rainbow response curve” that maps the hyperspectral response of the Polarsens‐based single‐shot *PolSpec* module.

SI Video V1 illustrates the dynamic changes in the SMV plot as the centre wavelength of the incident beam was swept from 450 to 650 nm. The white dashed line in the SMV plot of Figure 5 indicates the respective theoretical shape of the response curve. For this Polarsens‐based single‐shot implementation, it should be a parabola defined by $y=1-x^{2}$ for the spectral modulation functions $\left\{\cos\left(\Lambda \nu \right),\sin^{2}\left(\Lambda \nu \right)\right\}$, as $\sin^{2}\left(\Lambda \nu \right)=1-\cos^{2}\left(\Lambda \nu \right)$. The measured response curve overlaps well with this theoretical prediction.

### 3.2 Brightfield *PolSpec* Imaging of an H&E‐Stained Histological Section

**FIGURE 6:**
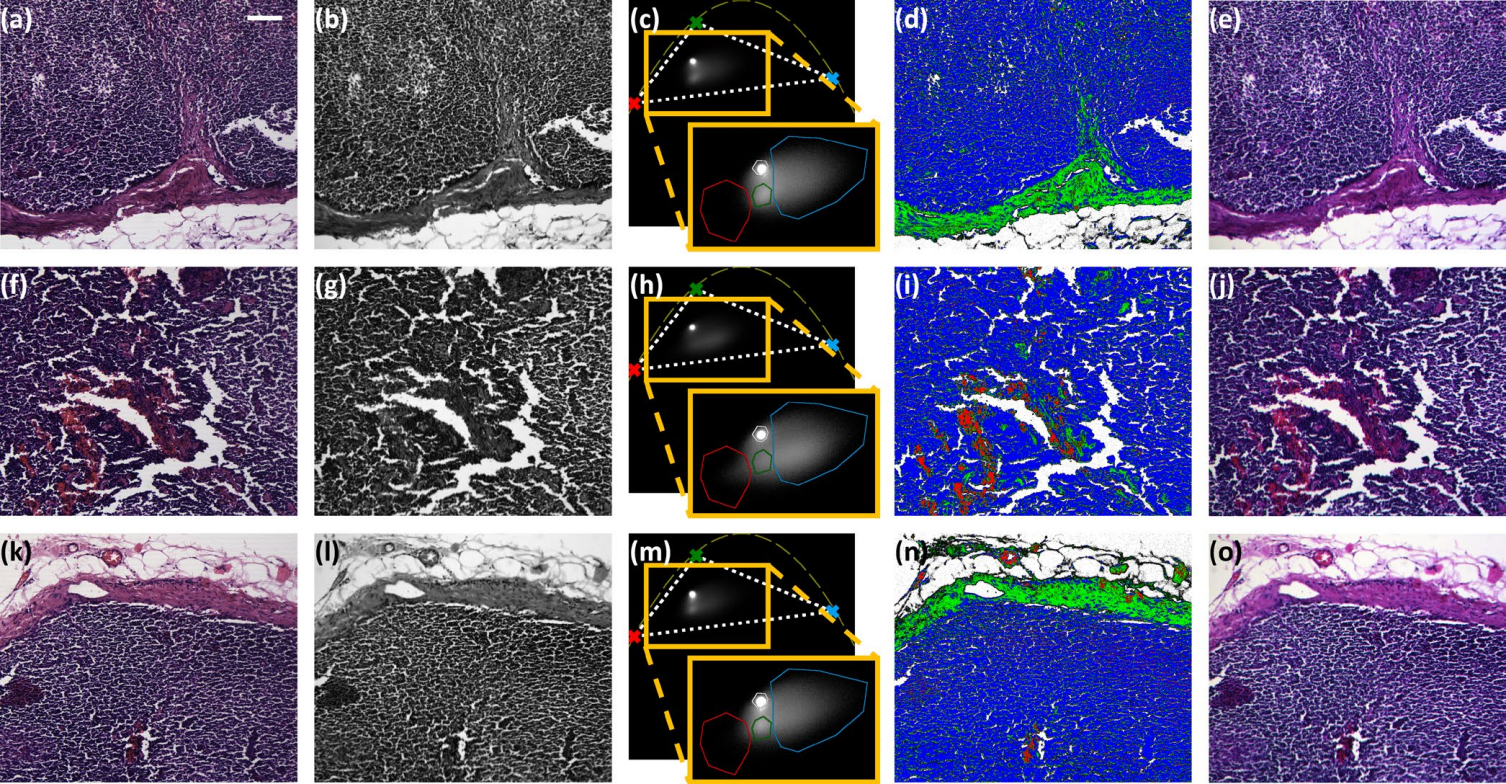
(a, f, k) RGB colour images from a H&E‐stained normal lymph node sample acquired by the colour CMOS camera white balanced to the illumination light from an unstained region. (b, g, l) are total intensity images acquired by the Polarsens‐based single‐shot *PolSpec* implementation of the same FOV as (a, f, k) and (e, j, o) are the corresponding pseudocolour images constructed with respect to the red, green and blue reference points (crosses) in the respective SMV plots shown in (c, h, m). The yellow dashed lines in the SMV plots indicate the theoretical IRF curves and the red, green and blue masks in the insets zoomed in delineate the pixels classified by the red, green and blue pixels shown in (d, i, n). (Scale bar: 100 μm).

The Polarsens‐based camera is well suited for brightfield imaging, and *PolSpec* can be configured to implement a colour (hyperspectral) camera in any spectral region within the sensitivity range of the camera. Here we demonstrate the application to imaging histological sections and validate this by directly comparing the results with those obtained using a conventional RGB CMOS camera in Figure 4. Figure 6 shows “colour” images from three different FOVs of a Haematoxylin and Eosin (H&E) stained human lymph node histological section that was transilluminated with white light from the white LED on the RGBW LED module in Figure 4.

From left to right in Figure 6, the first column in each row shows the RGB colour images of each FOV acquired by the colour camera, for which the white balance for this illumination was set up beforehand using its built‐in white balance functionality with a region showing no staining. The second and third columns in Figure 6 present the total intensity images and the respective SMV plots obtained from the Polarsens‐based single‐shot *PolSpec* image data.

Four masks were applied to the SMV plots, indicated by red, green, blue and white bounded areas in the orange insets of Figure 6c,h,m, and were used to identify spectrally similar pixels in the FOV to create the maps in the fourth column of Figure 6. Comparing the first and fourth columns in Figure 6, it can be observed that the regions classified as red, green and blue components by masks in the SMV plots coincide, respectively, with the red, pink and purple regions in the RGB colour images acquired by the colour camera, differentiating the capillary areas, the lymph node capsule and follicular regions. The pixels within the white mask in the SMV plots appear to correspond to background pixels in the FOV. This consistency with conventional colour camera images validates the capability of *PolSpec* to perform spectrally resolved imaging and indicates its potential for the classification of image components.

The last column in Figure 6 presents “RGB” pseudocolour images synthesised from the *PolSpec* image data. For each FOV, the synthesised RGB image was obtained by first decomposing the SMV at every pixel into three reference SMVs that were selected to represent the “pure” red, green and blue colours and then white balancing with respect to the illumination light for better visualisation contrast. The reference SMVs are indicated by red, green and blue crosses in the SMV plots of Figure 6c,h,m. Details about the synthesis and white balancing process are discussed in SI, and the impact of white balancing is illustrated in SI Figure S2.

The synthesised white‐balanced pseudocolour RGB images in Figure 6e,j,o look similar to those acquired by the colour CMOS camera in Figure 6a,f,k. Noting that differences in observed colour will occur, as the mosaic filters in front of the colour camera sensor have different spectral transmission profiles compared to the spectra corresponding to the references used for producing the pseudocolour images. These synthesised white‐balanced pseudocolour RGB images in Figure 6e,j,o may provide more information than the spectrally classified images in Figure 6d,i,n and could be helpful in identifying features of interest where there is no prior knowledge of spectral properties.

We note that, while here, the Polarsens‐based single‐shot *PolSpec* implementation has been directly compared to a colour camera in the visible range, a *PolSpec* module could be easily configured to provide “colour” imaging in a spectral region where no “RGB” type sensor (with mosaic filter) is available, for example, in the red/NIR spectral region, or could be configured to provide higher spectral resolution than the standard RGB mosaic filter. If single‐shot hyperspectral imaging outside the spectral sensitivity of a Polarsens‐based camera is required, a *PolSpec* configuration similar to that depicted in Figure 2 could be used. Alternatively, a simpler *PolSpec* configuration with two cameras, or an image splitter with one camera, could be implemented with an adjustable retarder (such as an LCR) and a two‐shot image data acquisition.

### 3.3 Fluorescence *PolSpec* Imaging of Pollen Grains

**FIGURE 7:**
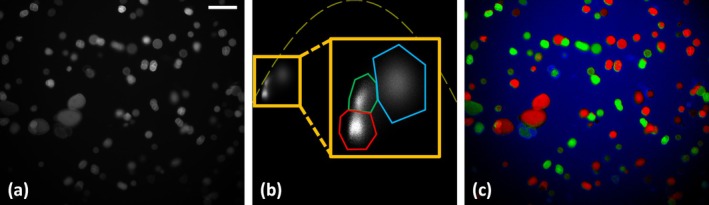
Fluorescence from a pollen grain sample acquired by the Polarsens‐based single‐shot *PolSpec* implementation. (a) is the total fluorescence intensity image of pollen grains and (b) is the SMV plot with yellow dashed line indicating the theoretical IRF curve and zoomed inset showing the regions of interest (masks) in the SMV used to classify the pixels and produce the colour‐coded total intensity image (c). (Scale bar: 100 μm).

*PolSpec* can also be implemented in fluorescence imaging instruments—with single‐shot hyperspectral imaging being attractive for dynamic samples, including in flow cytometers. As a proof of principle, Figure 7 demonstrates single‐shot *PolSpec* hyperspectral imaging of fluorescent pollen grains. With the passband of the supercontinuum spectral selection module set to produce 423–451 nm excitation and a long‐pass emission filter cutting off at 520 nm (Edmund Optics, Hoya Y52) installed before the input linear polariser of the *PolSpec* module, the fluorescent pollen grains were imaged. Figure 7a shows the total fluorescence intensity images and (b) presents the calculated SMV plots in which three distinct SMV clusters corresponding to different spectral components can be observed. Using the three masks indicated in Figure 7b by the red, green and blue bounded regions in the orange insets, every pixel in the FOV can be classified according to these spectral components, as shown in Figure 7c, where two “types” of pollen grains, coloured red and green respectively, are differentiated through their fluorescence spectral signatures. The blue component is shown to be background in the fluorescence image, which could be due to autofluorescence from the mounting media of the pollen grain sample.

## 4 Discussion

In principle, the spectral range and resolution of *PolSpec* implementations depend on the frequency Λ in the spectral modulation function(s), which is a function of the retardance. Using higher retardances will increase the spectral resolution but decrease the spectral range. This could be useful for applications requiring high spectral resolution within a limited spectral range, e.g., Raman spectroscopy.

In practise, the utility of a *PolSpec*‐based hyperspectral imaging module will also depend on the SNR of the detected signals, and it is generally desirable to be as photon efficient as possible. We note that the single‐shot *PolSpec* configuration implemented here requires a linear polariser at the input, which causes up to 50% loss for unpolarised incident light such as fluorescence and could be higher for inconveniently polarised incident light. Such input loss could potentially be minimised by placing a wave plate at the input and orienting it to maximise light transmitted through the linear polariser. However, it is possible to make *PolSpec* notionally lossless and insensitive to input polarisation by replacing the input linear polariser with a polarising beamsplitter and implementing two parallel *PolSpec* setups for the orthogonally polarised input components (as shown in Figure 2). This could also provide information concerning polarisation anisotropy, which could be useful for fluorescence imaging and sensing applications.

Aside from the specific spectral modulation functions, $\left\{\cos\left(k\Lambda \nu \right)\right\}$ where k=1,2,…, implemented in the work reported here, there are a wide range of other potential spectral modulation functions that can be realised using polarisation optics. As discussed in Methods, it is possible to implement conventional spectral phasor imaging, i.e., based on the spectral modulation functions $\left\{\cos\left(\Lambda \nu \right),\sin\left(\Lambda \nu \right)\right\}$, using *PolSpec* configurations incorporating AQWPs. However, these relatively exotic optical components may not always be available or cost‐effective. Two possible configurations for this are depicted in SI Figure S3 and Figure S4. Other sets of *PolSpec* spectral modulation functions such as $\left\{\cos\left(\Lambda \nu \right),\sin\left(\Lambda \nu \right)\sin\left(\Lambda \nu /X\right)\right\}$ where X is a positive integer, could also be realised, as shown in SI Figure S5. By carefully choosing X and Λ, the response curve of this configuration, as shown in SI Figure S6, can be similar to that of the standard phasor configuration within certain spectral range, but without requiring AQWPs.

In this work, we have focused on cost‐effective single‐shot implementations of SVM imaging utilising the Polarsens‐based polarisation resolving camera. The lack of cooling of such cameras will make them unsuitable for many fluorescence imaging applications, and there may be applications where the inevitable 50% loss of the mosaic polarisation filter that is integral to the sensor is unacceptable. An alternative strategy to the approaches depicted in Figures 2 and 3 is to use a polarisation‐resolving image splitter with a single camera and to acquire two sequential acquisitions with different retardances, as depicted in SI Figure S7. This approach can work with any detector, which may be selected for a particular spectral range of sensitivity, such as the mid‐IR, for high dynamic range of detection, or for high frame rate. The retardance could be adjusted by mechanical optical component changes or using an electronically tunable LCR. This confers many advantages for flexible operation, including the ability to tune the instrument for optimal detection of specific spectral features and to unmix more spectral components by applying more spectral modulation functions. SI Figure S8a,b shows the experimental implementation and corresponding “rainbow response curve” acquired by tuning the supercontinuum source (analogous to Figure 5) and (c–e) shows the application of the two‐shot LCR‐based PolSpec module applied to fluorescence imaging of the same pollen grain sample as in Figure 7. We note that an LCR has previously been used to implement spectral phasor imaging using a SWIR camera, as reported in arXiv [16], although the authors used a less efficient approach entailing the acquisition of three images with notionally cosine and sine spectral modulation functions and total transmission.

## 5 Conclusions

We have proposed *PolSpec*, a widefield hyperspectral imaging technique based on the direct acquisition of image data to calculate generalised SMVs by using polarisation optics to spectrally modulate incident light. *PolSpec* has the same advantages over conventional hyperspectral techniques as direct spectral phasor [9, 10] imaging, including a smaller data footprint, faster speed, better SNR, and the capability to measure diffuse (scattered) light signals, but *PolSpec* configurations may be cheaper to implement and more flexible in some situations, particularly if polarisation‐resolving cameras or electronically controlled LCRs are utilised. Here we demonstrated a compact single‐shot approach based on a polarisation‐resolving camera that could be used for rapid spectral classification, including dynamic samples. We note that a tuneable/adaptable implementation could be configured using an LCR, and we presented the first results from our implementation of this two‐shot *PolSpec* module.

*PolSpec* can, in principle, be implemented in almost any optical instrument using any detector where there are polarisation optics available in the appropriate spectral region. This could include wide‐field, light‐sheet, line‐scanning, and confocal/multiphoton laser scanning microscopes, endoscopes, telescopes, remote sensing systems, and even mobile phone cameras if the polarisation components could be miniaturised. For compact instruments, the spectral modulation functions could also be implemented using fibre‐optic‐based polarisation components.

*PolSpec* could be combined with fluorescence lifetime imaging (FLIM) to enable unmixing of more channels using both spectral and lifetime signatures, e.g., for highly multiplexed readouts of fluorescence labels as desired for some spatial proteomics applications. If applied to Förster resonance energy transfer (FRET) measurements, *PolSpec* could enable straightforward recording and unmixing of signals from both donor and acceptor fluorophores for more sophisticated and robust analysis. Some *PolSpec* configurations can also provide polarisation‐resolved measurements, e.g., to map fluorescence anisotropy as well as spectral properties.

Potential applications of *PolSpec*‐based multispectral and hyperspectral analysis include biomedical and physical sciences research, pathology, in vivo diagnostics (e.g., screening for abnormal tissue potentially indicating cancer), forensics, and multiplexed fluorescence readouts (e.g., for sequencing, spatial proteomics, immunofluorescence, etc.). It could also be applied to remote sensing for environmental monitoring, surveillance of plants (e.g., [16]) and (including crops from drones), and so on. As for other spectrally resolved imaging modalities, *PolSpec* image data can also be further combined with machine learning to enhance both spectral classification and unmixing applications.

## Author Contributions

**Huihui Liu:** conceptualisation, investigation, writing  –  original draft, writing  –  review and editing. **Sunil Kumar:** investigation, writing  –  review and editing. **Edwin Garcia:** resources, investigation, writing  –  review and editing. **Darren Ennis:** resources, investigation, writing  –  review and editing. **Iain A. McNeish:** resources, writing  –  review and editing. **Paul M. W. French:** conceptualisation, supervision, writing  –  original draft, writing  –  review and editing.

## Conflicts of Interest

*PolSpec* was presented at the European Microscopy Congress in Copenhagen, August 2024, and has been presented on bioRxiv [17]. The authors are working towards commercialising aspects of *PolSpec*.

## Supporting information


**Data S1.** Supporting Information.

**Video S1.** Instrument response function of Polarsens‐based single‐shot PolSpec module: video of the spectral modulation vector plot as input wavelength is tuned.

**Video S2.** Instrument response function of LCR‐based two‐shot PolSpec module: video of the spectral modulation vector plot as input wavelength is tuned.

## Data Availability

The data that support the findings of this study are openly available in Zenodo at https://doi.org/10.5281/zenodo.14771724.
