# Supplementary material for: PolSpec : Polarisation‐Based Detection for Versatile, Cost‐Effective Rapid Hyperspectral Imaging
Source: J Biophotonics. 2025 Mar 24;18(8):e70012. doi: 10.1002/jbio.70012 (PMC12318642; doi:10.1002/jbio.70012)
Supplement: Supplementary file 1 — Data S1. Supporting Information. [file JBIO-18-e70012-s003.docx]

***PolSpec*: polarisation-based detection for versatile, cost-effective rapid hyperspectral imaging**

**Supplementary Information**

**A. Hyperspectral imaging of a colour test chart using Polarsens™-based single-shot *PolSpec***

**To minimise costs, it is possible to implement a** Polarsens™**-based single-shot *PolSpec* configuration, following the same set-up as Figure 5(b) except that all polarisation optics used can be cut from** cheap off-the-shelf polymer polarising and retarder sheets (**Edmund Optics, XP42-40 & WP280**) at a total cost of less than £100. This implementation was applied to image a colour test chart [1] displayed on a mobile phone screen. **Figure S1(a) provides** the “ground truth” of colour distribution across the test chart image while Figure S1(b) shows the total intensity image and Figure S1**(c)** shows the SMV plot corresponding to three small regions selected from the red, green, and blue areas in the test chart, indicated in Figure S1**(b)**.

The three SMV point clouds in Figure S1**(c) indicate three different spectral components (“colours”) present in the test chart. Three masks drawn around these SMV point clouds - shown as dashed shaded regions in the inset of** Figure S1**(c) were then used to “classify” every pixel in the image FOV as red, green, or blue pixels.** Figure S1**(d) demonstrates the resulting intensity image, which is consistent with the colour ground truth in** Figure S1**(a). (Note that pixels not classified to any of three SMV regions were assigned to be white).**


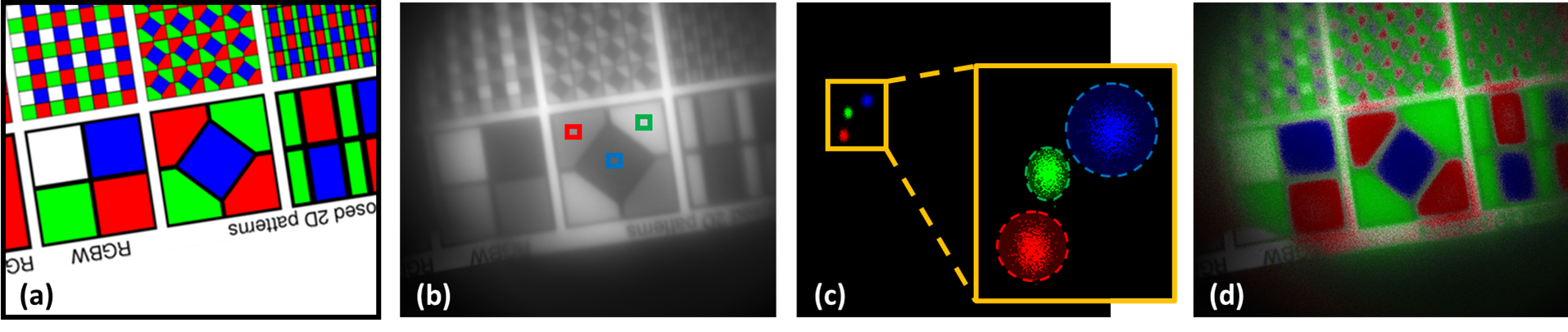


*Figure S1. Colour test chart imaged by a* ***Polarsens™-based single-shot PolSpec implementation:*** *(a) colour test chart ground truth, (b) total intensity image, (c) SMV plot of three bounded regions in (b), (d) colour-coded intensity image generated using masks derived from dashed shaded regions in the inset of (c).*

**B. Synthesis of *PolSpec* pseudocolour RGB images of H&E-stained lymph node including white balancing**

To synthesize **pseudocolour RGB images** from *PolSpec* data, the SMVs acquired were decomposed into three reference SMVs representing the “pure” red, green, and blue colours. This was achieved by solving the fraction $\alpha_{c}\left( x,y \right)$ of each reference SMV representing the colour $c$, where $c\in\left[ r,g,b \right]$, at every pixel $(x,y)$ in the image from linear equations:

|  | $\sum_{c} \alpha_{c}\left( x,y \right)\cdot V_{R,c}^{k}=V_{S}^{k}\left( x,y \right), k=1,\ldots,N$ | (S1) |
| --- | --- | --- |
|  | $\sum_{c} \alpha_{c}\left( x,y \right)=1$ | (S2) |

where ${\vec{\boldsymbol{V}}}_{S}\left( x,y \right)=\left( V_{S}^{1}\left( x,y \right),\ldots,V_{S}^{N}\left( x,y \right) \right)$ denotes the SMV acquired at the pixel $(x,y)$, ${\vec{\boldsymbol{V}}}_{R,c}=\left( V_{R,c}^{1},\ldots,V_{R,c}^{N} \right)$ denotes the reference SMV representing the colour $c$, and $N=2$ is the number of orthogonal spectral modulation functions used for this **Polarsens™-based single-shot *PolSpec* implementation**.

Here, **the red, green and blue reference SMVs** were chosen to **ensure that the area enclosed by three reference SMVs, indicated by white dotted triangles in the SMV plots of Figure 6(c,h,m), covers almost all SMVs of lymph node acquired from these FOVs.**

After decomposing, a pseudocolour RGB image can be generated by setting each pixel $(x,y)$ in the image to be

|  | $\left( R\left( x,y \right),G\left( x,y \right),B\left( x,y \right) \right)=\left( I_{tot}\left( x,y \right)\cdot\alpha_{r}\left( x,y \right), I_{tot}\left( x,y \right)\cdot\alpha_{g}\left( x,y \right), I_{tot}\left( x,y \right)\cdot\alpha_{b}\left( x,y \right) \right)$ | (S3) |
| --- | --- | --- |

where $I_{tot}\left( x,y \right)$ is the total intensity at the pixel $(x,y)$.

However, such a pseudocolour RGB image is not white balanced with respect to the illumination light from the white LED and therefore differences in the relative strengths of the RGB components of the illumination could change the colour contrast of these images to human vision, as demonstrated in **Figure S2**, which could impact interpretation and classification. To improve visualization, the SMV of the white LED illuminated light background (at an unstained region) was acquired using the *PolSpec* module and then decomposed with respect to the same three reference SMVs to obtain the weightings $\left( \alpha_{r}^{w},\alpha_{g}^{w},\alpha_{b}^{w} \right)$ of each reference required to achieve white-balancing for the white LED illumination light. These weights were used to produce the white balanced pseudocolour RGB images presented in Figure S2(d-f) (equivalent to Figure 6(e,j,o)) by scaling the intensity of each pixel $(x,y)$ in the image:

|  | $\left( R^{WB}\left( x,y \right),G^{WB}\left( x,y \right),B^{WB}\left( x,y \right) \right)=\left( \frac{R\left( x,y \right)}{\alpha_{r}^{w}},\frac{G\left( x,y \right)}{\alpha_{g}^{w}},\frac{B\left( x,y \right)}{\alpha_{b}^{w}} \right)$ | (S4) |
| --- | --- | --- |

Figure S2 **demonstrates the pseudocolour RGB images of H&E-stained lymph node in the same three FOVs as shown in Figure 6, which were synthesized without (a-c) and with (d-f) white balancing with respect to the white transillumination light.**


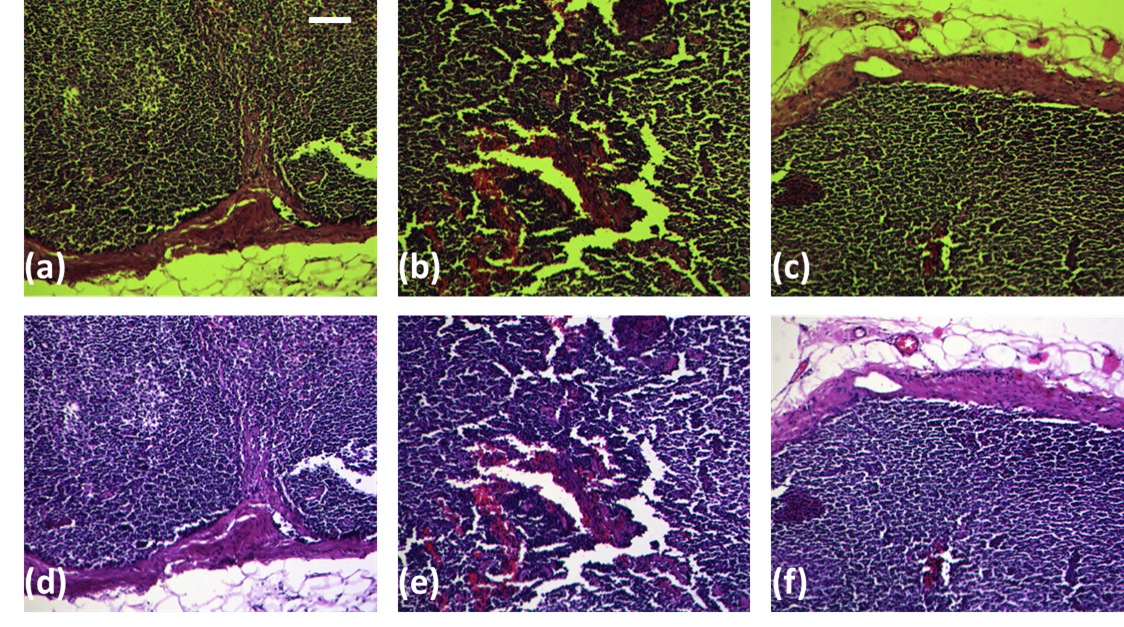


*Figure S2. Synthesized pseudocolour RGB images from PolSpec SMV data of H&E-stained lymph node (a-c) before and (d-f) after white balancing. (d-f) are same as Figure 6(e,j,o). (Scale bar: 100 µm)*

**C. *PolSpec* configurations with different spectral modulation functions**

*C-1. Spectral phasor modulation functions implemented with achromatic quarter wave plates*

**Figure S3** and **Figure S4** depict two *PolSpec* configurations for generating spectral phasors, i.e., SMVs corresponding to the classical spectral phasor modulation functions, $\left\{ \cos\left( \Lambda\nu\right),\sin\left( \Lambda\nu\right) \right\}$, by incorporating achromatic quarter wave plates (AQWPs) to provide the $\sin\left( \Lambda\nu\right)$ modulation function. While the configuration in Figure S3 is analogous to SHy-Cam [2] and quite complicated to implement, the configuration in Figure S4 based on a polarisation-resolving (Polarsens™) camera is more compact. **Equation** **(S5)**-**(S7)** and **Equation** **(S8)**-**(S10)** present the derivations of SMVs for the configurations in Figure S3 and Figure S4, respectively.

For the Figure S3 configuration:

|  | $I_{0,0}=\int\frac{I_{i0}(\nu)}{2}\left[ 1+\cos\left( \Lambda\nu\right) \right]\cdot d\nu, I_{0,90}=\int\frac{I_{i0}(\nu)}{2}\left[ 1-\cos\left( \Lambda\nu\right) \right]\cdot d\nu$ | (S5) |
| --- | --- | --- |
|  | $I_{90,0}=\int\frac{I_{i90}(\nu)}{2}\left[ 1+\sin\left( \Lambda\nu\right) \right]\cdot d\nu, I_{90,90}=\int\frac{I_{i90}(\nu)}{2}\left[ 1-\sin\left( \Lambda\nu\right) \right]\cdot d\nu$ | (S6) |
|  | $\vec{\boldsymbol{V}}=\left( \frac{I_{0,0}-I_{0,90}}{I_{0,0}+I_{0,90}},\frac{I_{90,0}-I_{90,90}}{I_{90,0}+I_{90,90}} \right)=\left( \frac{\int I_{i0}\left( \nu\right)\cdot\cos\left( \Lambda\nu\right)\cdot d\nu}{\int I_{i0}\left( \nu\right)\cdot d\nu},\frac{\int I_{i90}\left( \nu\right)\cdot\sin\left( \Lambda\nu\right)\cdot d\nu}{\int I_{i90}\left( \nu\right)\cdot d\nu} \right)$ | (S7) |


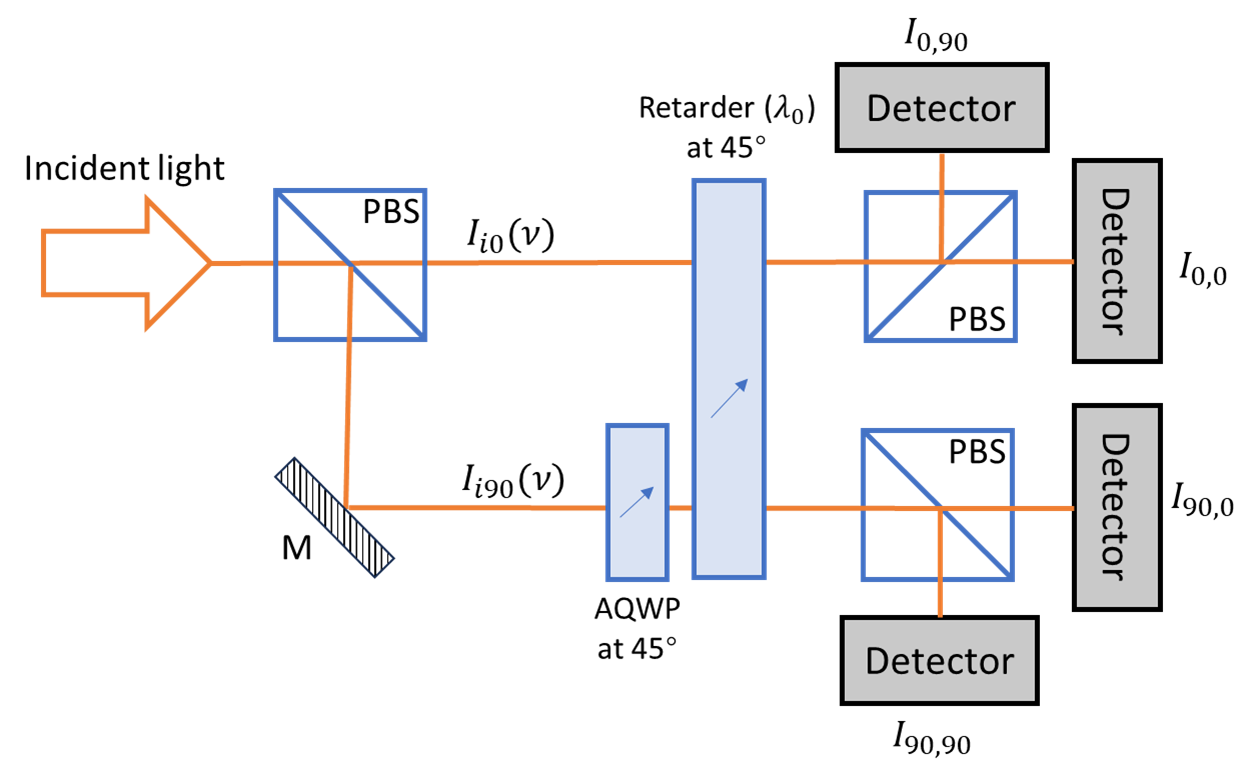


*Figure S3. Single-shot PolSpec for spectral phasors with* $\left\{ \cos\left( \Lambda\nu\right),\sin\left( \Lambda\nu\right) \right\}$ *spectral modulation functions.*


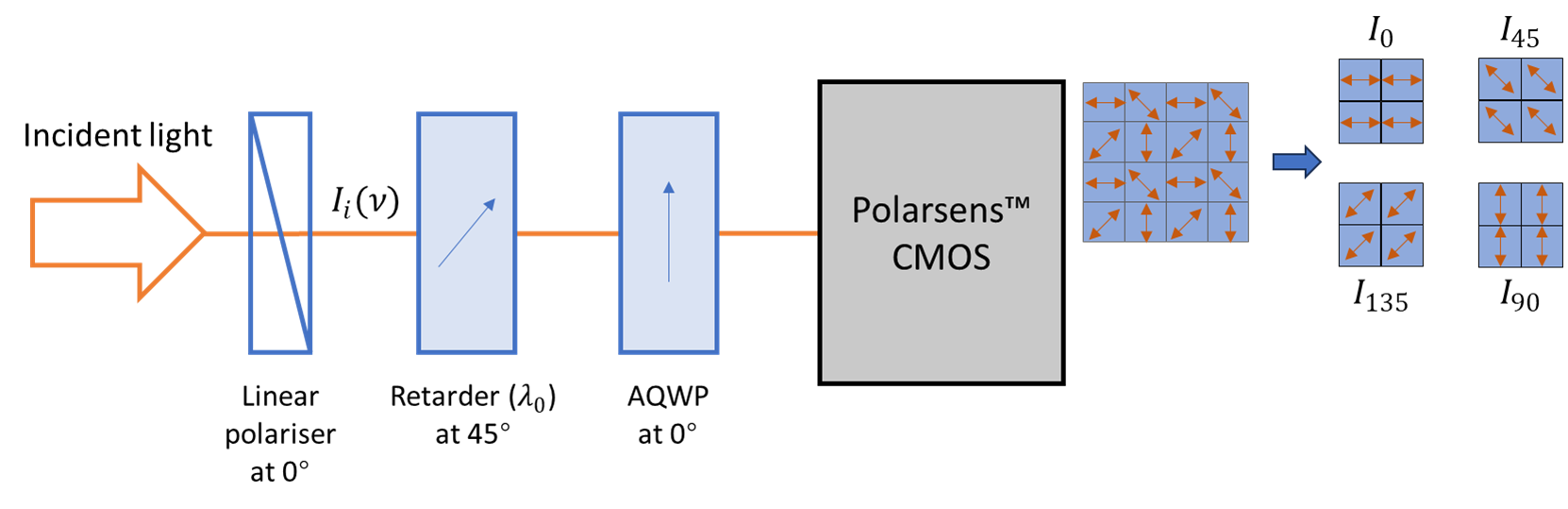


*Figure S4. Polarsens™-based single-shot PolSpec for spectral phasors with* $\left\{ \cos\left( \Lambda\nu\right),\sin\left( \Lambda\nu\right) \right\}$ *spectral modulation functions.*

For the Figure S4 configuration:

|  | $I_{0}=\int\frac{I_{i}\left( \nu\right)}{2}\left[ 1+\cos\left( \Lambda\nu\right) \right]\cdot d\nu, I_{90}=\int\frac{I_{i}\left( \nu\right)}{2}\left[ 1-\cos\left( \Lambda\nu\right) \right]\cdot d\nu$ | (S8) |
| --- | --- | --- |
|  | $I_{45}=\int\frac{I_{i}\left( \nu\right)}{2}\left[ 1+\sin\left( \Lambda\nu\right) \right]\cdot d\nu, I_{135}=\int\frac{I_{i}\left( \nu\right)}{2}\left[ 1-\sin\left( \Lambda\nu\right) \right]\cdot d\nu$ | (S9) |
|  | $\vec{\boldsymbol{V}}=\left( \frac{I_{0}-I_{90}}{I_{0}+I_{90}},\frac{I_{45}-I_{135}}{I_{45}+I_{135}} \right)=\left( \frac{\int I_{i}\left( \nu\right)\cdot\cos\left( \Lambda\nu\right)\cdot d\nu}{\int I_{i}\left( \nu\right)\cdot d\nu},\frac{\int I_{i}\left( \nu\right)\cdot\sin\left( \Lambda\nu\right)\cdot d\nu}{\int I_{i}\left( \nu\right)\cdot d\nu} \right)$ | (S10) |

*C-2. Phasor-like modulation vectors not requiring an achromatic quarter wave plate*

**Figure S5** presents a Polarsens™-based single-shot configuration with a different set of spectral modulation functions, $\left\{ \cos\left( \Lambda\nu\right),\sin\left( \Lambda\nu\right)\sin\left( \Lambda\nu/X \right) \right\}$ where $X\in\mathbb{N}^{+}$, and the SMV derivation process for this configuration is shown in **Equation** **(S11)**-**(S13)**.

|  | $I_{0}=\int\frac{I_{i}\left( \nu\right)}{2}\left[ 1+\cos\left( \Lambda\nu\right) \right]\cdot d\nu, I_{90}=\int\frac{I_{i}\left( \nu\right)}{2}\left[ 1-\cos\left( \Lambda\nu\right) \right]\cdot d\nu$ | (S11) |
| --- | --- | --- |
|  | $I_{45}=\int\frac{I_{i}\left( \nu\right)}{2}\left[ 1+\sin\left( \Lambda\nu\right)\sin\left( \frac{\Lambda\nu}{X} \right) \right]\cdot d\nu, I_{135}=\int\frac{I_{i}\left( \nu\right)}{2}\left[ 1-\sin\left( \Lambda\nu\right)\sin\left( \frac{\Lambda\nu}{X} \right) \right]\cdot d\nu$ | (S12) |
|  | $\vec{\boldsymbol{V}}=\left( \frac{I_{0}-I_{90}}{I_{0}+I_{90}},\frac{I_{45}-I_{135}}{I_{45}+I_{135}} \right)=\left( \frac{\int I_{i}\left( \nu\right)\cdot\cos\left( \Lambda\nu\right)\cdot d\nu}{\int I_{i}\left( \nu\right)\cdot d\nu},\frac{\int I_{i}\left( \nu\right)\cdot\sin\left( \Lambda\nu\right)\cdot\sin\left( \Lambda\nu/X \right)\cdot d\nu}{\int I_{i}\left( \nu\right)\cdot d\nu} \right)$ | (S13) |


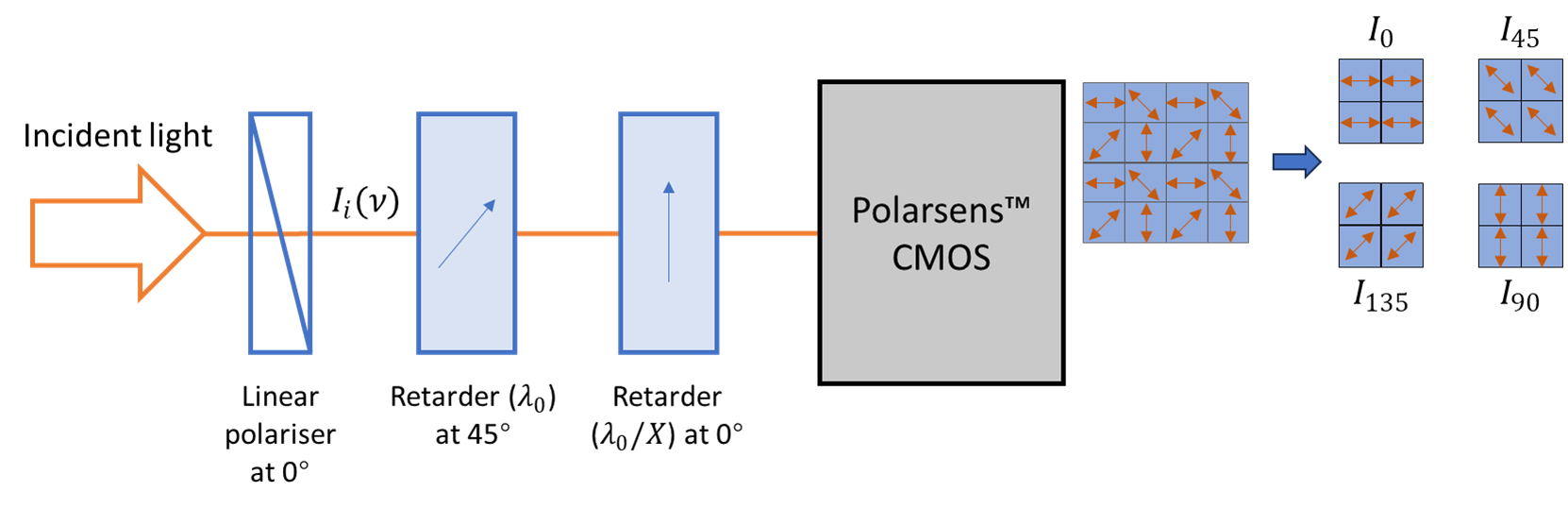


*Figure S5. Polarsens™-based single-shot PolSpec with* $\left\{ \cos\left( \Lambda\nu\right),\sin\left( \Lambda\nu\right)\sin\left( \Lambda\nu/X \right) \right\}$ *spectral modulation functions.*

**Figure S6** presents the theoretical IRF curves for the classical spectral phasor modulation functions $\left\{ \cos\left( \Lambda\nu\right),\sin\left( \Lambda\nu\right) \right\}$ and for two specific implementations of the configuration shown in Figure S5. These two specific implementations provide theoretical IRF curves close to that of the spectral phasor configuration within a limited spectral range but without requiring AQWPs in the set-up.


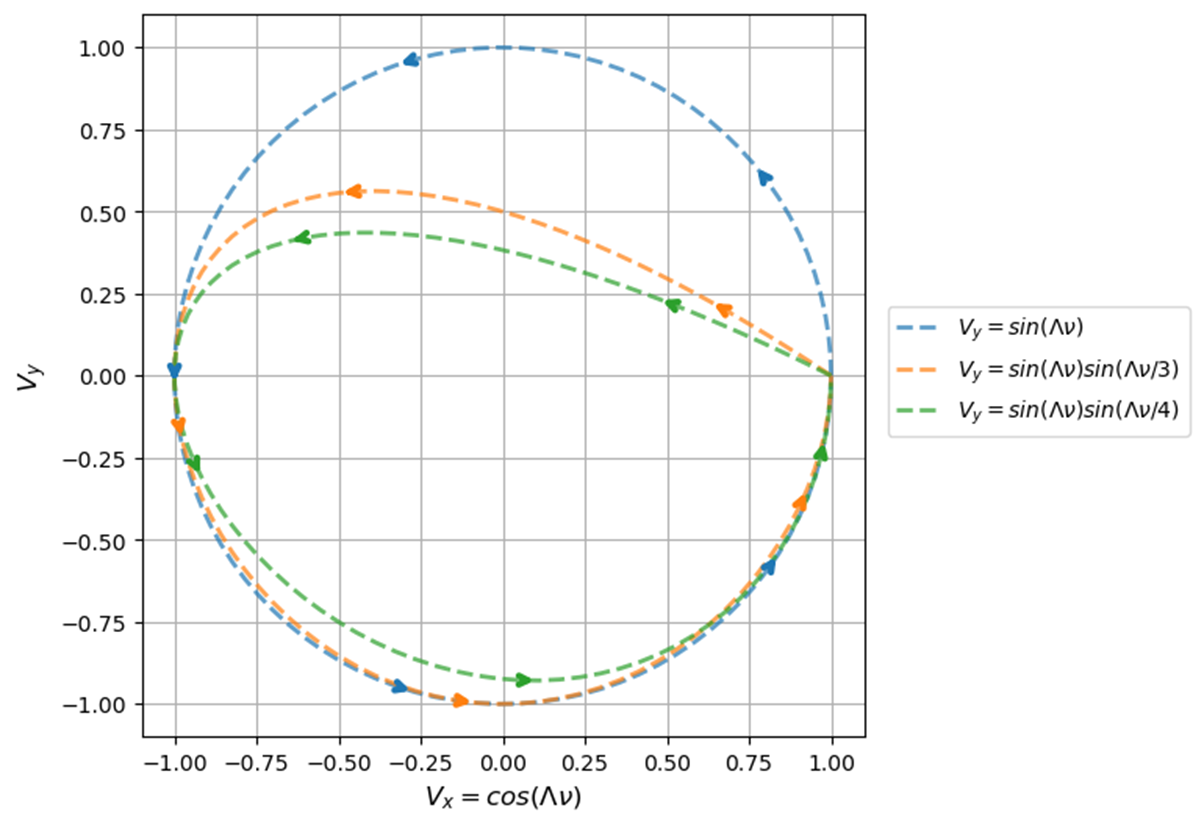


*Figure S6. Theoretical IRF curves of PolSpec configurations with various sets of spectral modulation functions:* $\{\cos\left( \Lambda\nu\right),\sin\left( \Lambda\nu\right)\}$ *(blue), and* $\{\cos\left( \Lambda\nu\right),\sin\left( \Lambda\nu\right)\sin\left( \Lambda\nu/X \right)\}$ *where* $X=3$ *(orange) and* $X=4$ *(green). Arrows indicate how the IRF curves vary with the value of* $\Lambda\nu$ *from 0 to 2π.*

**D. LCR-based *PolSpec* configuration**

**Figure S7** depicts a *PolSpec* configuration that is compatible with a range of imaging modalities, including fluorescence microscopy. An LCR is placed with its (extra)ordinary axis oriented at 45° to the fast axis of input polariser. The output from the LCR comprises two orthogonally linearly polarised components that are detected separately as the complement of each other. By electronically varying the retardance of the LCR to be integral multiples of some $\lambda_{0}$ and acquiring the complementary readouts for each retardance value, $N$ dimensional SMVs with the spectral modulation functions, $\left\{ \cos\left( k\Lambda\nu\right) \right\}$ where $k=1,2,\ldots,N$, can be generated.

When the retardance of the LCR is $k\lambda_{0}$, two complementary readouts detected can be written as

|  | $I_{0}=\int\frac{I_{i}\left( \nu\right)}{2}\left[ 1+\cos\left( k\Lambda\nu\right) \right]\cdot d\nu, I_{90}=\int\frac{I_{i}\left( \nu\right)}{2}\left[ 1-\cos\left( k\Lambda\nu\right) \right]\cdot d\nu$ | (S14) |
| --- | --- | --- |

Therefore, the $k$-th elements in the SMVs can be calculated by

|  | $V_{k}=\frac{I_{0}-I_{90}}{I_{0}+I_{90}}=\frac{\int I_{i}\left( \nu\right)\cdot\cos\left( k\Lambda\nu\right)\cdot d\nu}{\int I_{i}\left( \nu\right)\cdot d\nu}$ | (S15) |
| --- | --- | --- |

In practical implementations, the number of spectral modulation functions, $N$ is usually two but can be increased as needed, e.g., to unmix more spectral components. The upper limit of $N$ is determined by the maximum retardance the LCR can reach, although this could be raised by adding additional fixed or variable retarders. With multiple image acquisitions there will be a trade-off with the imaging speed, although we note that LCR can be switched at >100 Hz rates and so *PolSpec* imaging rates of 10’s Hz can be realised.

We note that, compared to the detection in the Polarsens™-based configuration, this is notionally lossless after the first polariser and can be implemented with a wide range of detectors as required by the specific imaging modality. For example, a two-channel polarisation imaging splitter (PIS) combined with a TE-cooled (sCMOS) camera could be implemented for wide-field fluorescence. For multiphoton or confocal, scanning microscopy, single pixel detectors such as photomultipliers or line detectors such as CCD arrays could also be incorporated with a polarisation beamsplitter (PBS) for *PolSpec*-based hyperspectral imaging.


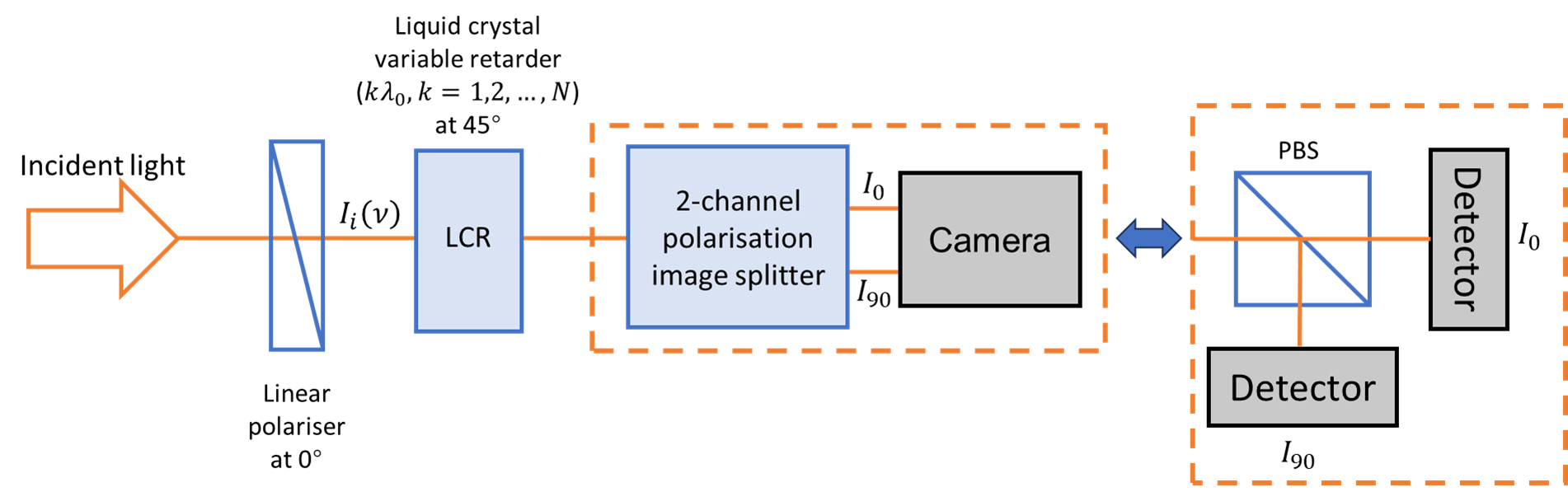


*Figure S7. LCR-based PolSpec with* $\left\{ \cos\left( k\Lambda\nu\right) \right\}$*, where* $k=1,2,\ldots N$*, spectral modulation functions.*

We have implemented an LCR-based *PolSpec* module following the configuration in Figure S7 and mounted it to the same camera port of the Olympus IX71 microscope frame as in **Figure 4**(a). The diagram of this LCR-based *PolSpec* module is depicted in **Figure S8**(a). T**he same input linear polariser, LP, and relay lenses, RL1 and RL2, were used and a full-wave LCR (Thorlabs,** LCC1423-A**) was placed immediately after RL2. A commercial PIS (**Optical Insights, Inc, Dual-View™**) was mounted in front of a TE-cooled CMOS camera (Cairn Research Ltd, CellCam Kikker 100MT) to serve as the detector.**

**For the results presented here, the LCR-based implementation was set to acquire 2D SMV image data corresponding to the** $\left\{ \cos\left( \Lambda\nu\right),\cos\left( 2\Lambda\nu\right) \right\}$ **spectral modulation functions in two sequential acquisitions: we set the LCR retardances to ~700 nm and ~350 nm respectively, by setting the AC signal applied to the LCR to RMS voltages of 1 V and 1.7 V, at a frequency of 2 kHz.**

**The instrument response function (IRF) of this LCR-based two-shot *PolSpec* implementation, as shown in** Figure S8(b), **was measured between** 450 nm and 650 nm following the same procedure used to generate Figure 5. **Supplementary Video V2** further demonstrate the dynamic changes in the SMV plot as the centre wavelength of the incident beam was swept from 450 nm to 650 nm. For this LCR-based two-shot implementation, the theoretical IRF, indicated by the white dashed line in Figure S8(b), should be also a parabola, but one defined by $y=2x^{2}-1$ **for** the spectral modulation functions $\left\{ \cos\left( \Lambda\nu\right),\cos\left( 2\Lambda\nu\right) \right\}$**, as** $\cos\left( 2\Lambda\nu\right)=2\cos^{2} \left( \Lambda\nu\right)-1$**.**

**It can be observed that there** is small difference between the measured and theoretical curves for this LCR-based two-shot implementation, which could have been caused by the actual retardances of the LCR being different from the theoretical values (which were interpolated from the LCR datasheet), or due to insufficient extinction between two channels of the polarisation image splitter. Nevertheless, the SMVs generated by this *PolSpec* implementation are still suitable for application such as spectral classification of fluorescence images. For proof of concept, the same fluorescent pollen grain sample was imaged using this LCR-based two-shot *PolSpec* module with the same excitation band and emission filter utilised for producing **Figure 7**. The results shown in Figure S8(c-e) demonstrate two types of pollen grains with different fluorescent spectral signatures being separated by masking in the SMV plot, which is consistent with the results in Figure 7.


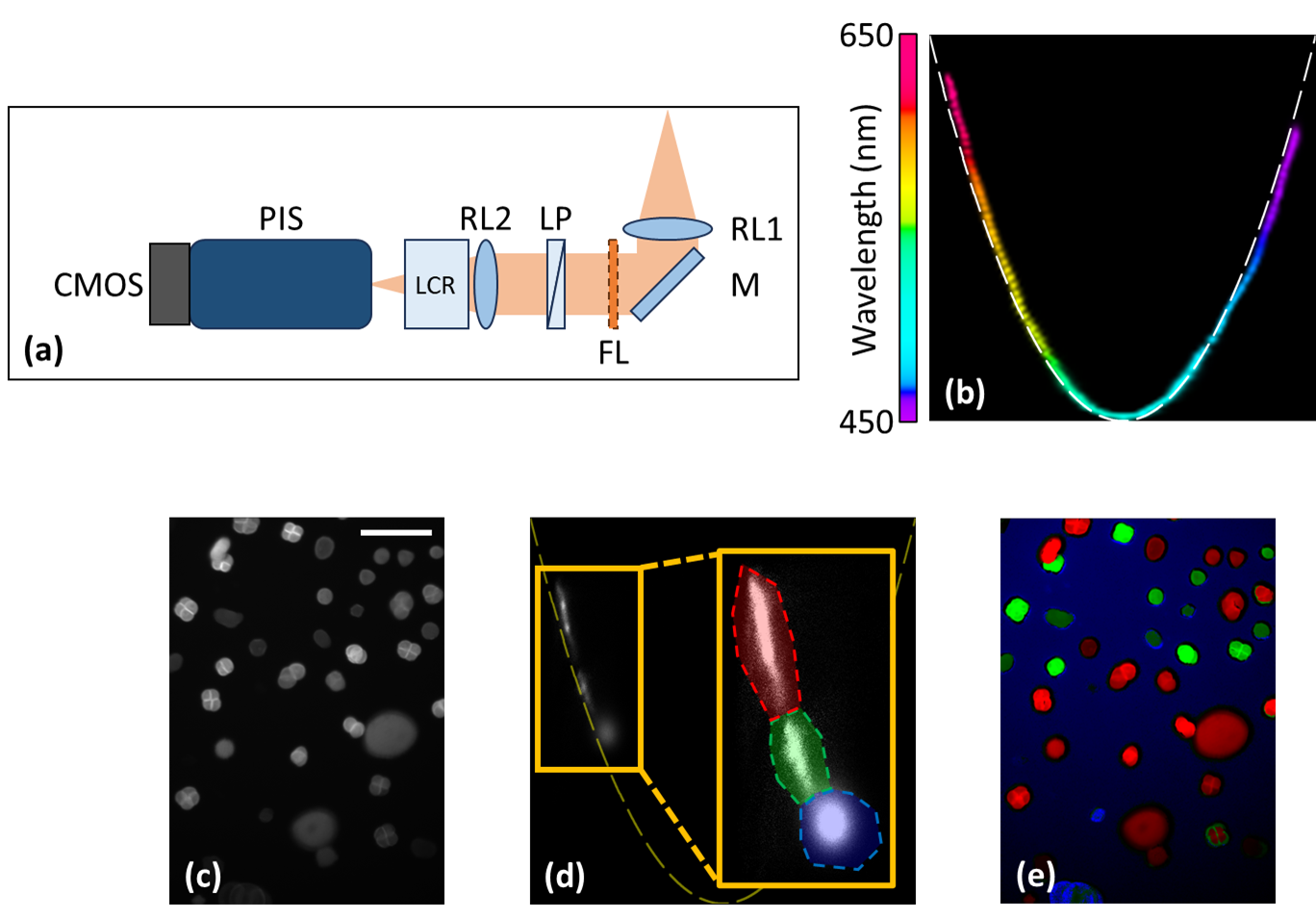


*Figure S8. (a) System diagram of the LCR-based PolSpec module implemented with a PIS and a sCMOS camera. (M, steering mirror; RL1 & RL2, relay lenses* *of 100 mm focal lengths; FL, emission filter for fluorescence imaging; LP, linear polariser; RT_45_ & RT_0_, retarders with extraordinary axis oriented at 45*° *& 0*°*)*

*(b) “Rainbow response curve” mapping the hyperspectral IRF of the LCR-based two-shot PolSpec module in (a) between 450 nm and 650 nm. White dashed line indicates the theoretical IRF curve for the PolSpec configuration with* $\left\{ \cos\left( \Lambda\nu\right),\cos\left( 2\Lambda\nu\right) \right\}$ *spectral modulation functions.*

*(c-e) Fluorescence from a pollen grain sample acquired by the PolSpec implementation in (a): From left to right are (c) the total fluorescence intensity image, (d) the SMV plot with the yellow dashed line indicating the theoretical IRF curve and the zoomed inset of the regions of interest used to colour (e) the colour-coded total intensity image masked by the dashed shaded regions in the inset of the SMV plot. (Scale bar: 100 µm).*

**References**

1. L. J. Kerofsky and D. S. Messing, "15.2: Optimal rendering for Colour Matrix Displays," (2005).

2. P. Wang, M. Kitano, K. Keomanee-Dizon, T. V. Truong, S. E. Fraser, and F. Cutrale, "A single-shot hyperspectral phasor camera for fast, multi-color fluorescence microscopy," Cell Reports Methods **3**(4), (2023).
